# Time Domains of Hypoxia Responses and -Omics Insights

**DOI:** 10.3389/fphys.2022.885295

**Published:** 2022-08-08

**Authors:** James J. Yu, Amy L. Non, Erica C. Heinrich, Wanjun Gu, Joe Alcock, Esteban A. Moya, Elijah S. Lawrence, Michael S. Tift, Katie A. O'Brien, Jay F. Storz, Anthony V. Signore, Jane I. Khudyakov, William K. Milsom, Sean M. Wilson, Cynthia M. Beall, Francisco C. Villafuerte, Tsering Stobdan, Colleen G. Julian, Lorna G. Moore, Mark M. Fuster, Jennifer A. Stokes, Richard Milner, John B. West, Jiao Zhang, John Y. Shyy, Ainash Childebayeva, José Pablo Vázquez-Medina, Luu V. Pham, Omar A. Mesarwi, James E. Hall, Zachary A. Cheviron, Jeremy Sieker, Arlin B. Blood, Jason X. Yuan, Graham R. Scott, Brinda K. Rana, Paul J. Ponganis, Atul Malhotra, Frank L. Powell, Tatum S. Simonson

**Affiliations:** ^1^ Division of Pulmonary, Critical Care and Sleep Medicine, Department of Medicine, School of Medicine, University of California, San Diego, La Jolla, CA, United States; ^2^ Department of Anthropology, Division of Social Sciences, University of California, San Diego, La Jolla, CA, United States; ^3^ Division of Biomedical Sciences, School of Medicine, University of California, Riverside, CA, United States; ^4^ Herbert Wertheim School of Public Health and Longevity Sciences, University of California, San Diego, La Jolla, CA, United States; ^5^ Department of Emergency Medicine, University of New Mexico, Albuquerque, MX, United States; ^6^ Department of Biology and Marine Biology, College of Arts and Sciences, University of North Carolina Wilmington, Wilmington, NC, United States; ^7^ Department of Physiology, Development and Neuroscience, Faculty of Biology, School of Biological Sciences, University of Cambridge, Cambridge, ENG, United Kingdom; ^8^ School of Biological Sciences, College of Arts and Sciences, University of Nebraska-Lincoln, Lincoln, IL, United States; ^9^ Department of Biological Sciences, University of the Pacific, Stockton, CA, United States; ^10^ Department of Zoology, Vancouver, BC, Canada; ^11^ Lawrence D. Longo, MD Center for Perinatal Biology, Loma Linda, CA, United States; ^12^ Department of Anthropology, Cleveland, OH, United States; ^13^ Laboratorio de Fisiología Comparada/Fisiología del Transporte de Oxígeno, Lima, Peru; ^14^ Department of Pediatrics, San Diego, CA, United States; ^15^ School of Medicine, University of Colorado Anschutz Medical Campus, Aurora, CO, United States; ^16^ Division of Reproductive Sciences, Department of Obstetrics and Gynecology, Aurora, CO, United States; ^17^ Department of Kinesiology, Southwestern University, Georgetown, TX, United States; ^18^ San Diego Biomedical Research Institute, San Diego, CA, United States; ^19^ Department of Medicine, UC San Diego School of Medicine, San Diego, CA, United States; ^20^ Department of Archaeogenetics, Max Planck Institute for Evolutionary Anthropology, Leipzig, Germany; ^21^ Department of Integrative Biology, College of Letters and Science, University of California, Berkeley, Berkeley, CA, United States; ^22^ Division of Pulmonary and Critical Care Medicine, Department of Medicine, School of Medicine, Johns Hopkins Medicine, Baltimore, MD, United States; ^23^ Division of Biological Sciences, College of Humanities and Sciences, University of Montana, Missoula, MT, United States; ^24^ Department of Pediatrics Division of Neonatology, School of Medicine, Loma Linda University, Loma Linda, CA, United States; ^25^ Department of Pediatrics Division of Neonatology, School of Medicine, Loma Linda University, Loma Linda, CA, United States; ^26^ Moores Cancer Center, UC San Diego, La Jolla, CA, United States; ^27^ Department of Psychiatry, UC San Diego, La Jolla, CA, United States; ^28^ Center for Marine Biotechnology and Biomedicine, La Jolla, CA, United States

**Keywords:** hypoxia, adaptation, high altitude, oxygen, integrative physiology

## Abstract

The ability to respond rapidly to changes in oxygen tension is critical for many forms of life. Challenges to oxygen homeostasis, specifically in the contexts of evolutionary biology and biomedicine, provide important insights into mechanisms of hypoxia adaptation and tolerance. Here we synthesize findings across varying time domains of hypoxia in terms of oxygen delivery, ranging from early animal to modern human evolution and examine the potential impacts of environmental and clinical challenges through emerging multi-omics approaches. We discuss how diverse animal species have adapted to hypoxic environments, how humans vary in their responses to hypoxia (i.e., in the context of high-altitude exposure, cardiopulmonary disease, and sleep apnea), and how findings from each of these fields inform the other and lead to promising new directions in basic and clinical hypoxia research.

## 1 Introduction

In parallel with the evolution of aerobic metabolism, various organisms evolved mechanisms for adapting to decreased oxygen (O_2_) availability. These alterations can be categorized by time domains ranging from gradual changes in atmospheric O_2_ concentrations over millions of years to immediate physiological responses upon exposure to hypoxic stress. In this review, we discuss adaptations and responses to hypoxia that span 1) millions of years of non-human animal evolution, 2) hundreds of generations since modern humans have occupied high altitudes, 3) physiological and epigenetic changes within a lifespan, and 4) physiologic and pathophysiologic hypoxic challenges. We highlight how different species have adapted to reduced O_2_ availability and how humans exhibit variable individual capacities to alter hypoxia pathway responses. We approach these topics from multiple perspectives and emphasize the importance of integrating physiological and -omics effects of hypoxia for future progress in biological and medical applications.

## 2 Time Domain 1: Adaptation to Hypoxia Across Non-Human Species

### 2.1 Oxygen Over Geologic Time and its Impact on Early Animal Pathway Evolution

Since the emergence of eukaryotes and multicellular life, Earth’s atmospheric and oceanic O_2_ levels have been in constant flux. It is estimated that atmospheric O_2_ ranged between 15 and 30% over the last 550 million years (Berner, 1999). During this time, most of the animal phyla we know today emerged and evolved, adapting to changes in O_2_ availability. Many major events in the evolutionary history of life coincided with rising atmospheric O_2_ levels (Falkowski et al., 2005; Berner et al., 2007; Mills et al., 2014).

Key hypoxia sensing and response pathways evolved to ensure prompt physiological responses when O_2_ demand exceeded supply. For example, the hypoxia-inducible factor (HIF) pathway coordinates expression of thousands of genes in response to hypoxia (Semenza, 2020) and is highly conserved with a majority of metazoans expressing HIF homologues (Hampton-Smith and Peet, 2009). The HIF pathway evolved in the common ancestor of Bilateria, Cnidaria, and Placozoa, as modern representatives of earlier-branching metazoan lineages (Ctenophores and Poriferans) lack the ability to respond to changes in O_2_ *via* transcriptional regulation (Mills et al., 2018). Once the HIF pathway was established, it underwent changes in complexity as O_2_ requirements became more demanding in larger animals and tissue metabolic requirements became specialized (Taylor and McElwain, 2010).

### 2.2 Physiological Adaptations to Hypoxia in Tetrapods

Comparative studies provide empirical generalizations about the nature of hypoxia adaptations and yield unique insight into adaptive mechanisms that would otherwise remain unknown. Air-breathing animals adapt to high-altitude hypoxia through physiological adjustments that sustain O_2_ flux to tissue mitochondria and thereby support aerobic ATP production. The first physiological response to hypoxemia is to increase ventilation to minimize the decline in the arterial partial pressure of O_2_ (P_O2_), which occurs within the first minutes of hypoxia exposure through the hypoxic ventilatory response (Teppema and Dahan, 2010; Ivy and Scott, 2015) (further discussed in Section 4). Studies of deer mice reveal that evolutionary adaptation to high altitude changes the hypoxic ventilatory chemoreflex to further increase breathing and pulmonary O_2_ uptake while attenuating sympathoadrenal activation (Scott et al., 2019; Storz and Scott, 2019). Highland deer mice maintain higher rates of alveolar ventilation and preserve ventilatory sensitivity to CO_2_ relative to lowland conspecifics (Ivy and Scott, 2017, 2018). Known exceptions to this common increase in ventilation are the naked mole rat and guinea pig (Gonzalez-Obeso et al., 2017). The naked mole rat is an extremely hypoxia-tolerant mammal that lives underground and has been shown to decrease ventilation upon hypoxia (Pamenter et al., 2015).

Changes in lung structure and function also represent important adaptations to chronic hypoxia at high altitude. Hypoxia during early life can impair septation (the partitioning of saccules into alveoli) and thus impede lung development (Blanco et al., 1991; Ambalavanan et al., 2008). These effects are overcome in high-altitude deer mice (West C. M. et al., 2021), whereby they and many other high-altitude animals exhibit larger lungs and/or higher alveolar surface density than low-altitude counterparts (Pearson and Pearson, 1976; Lechner, 1977; Maina et al., 2017). High-altitude deer mice are also more effective at ventilation-perfusion matching in chronic hypoxia than low-altitude mice (West CM. et al., 2021). These changes help increase the morphological and physiological capacities for O_2_ diffusion of the lungs, and thus help augment arterial P_O2_ and O_2_ saturation in chronic hypoxia.

Another adaptive mechanism to improve arterial O_2_ saturation is achieved through genetically based changes in hemoglobin (Hb), the protein responsible for circulatory O_2_ transport (Storz, 2016, 2019). Many vertebrates native to high altitude have evolved increased Hb-O_2_ affinity (Storz, 2016) and, in some cases, the adaptive protein modification helps safeguard arterial O_2_ saturation under severe hypoxia (Tate et al., 2017, 2020; Storz and Bautista, 2021). These changes are attributed to amino acid replacements at numerous sites in the *a*- and *ß*-chain subunits of the α_2_β_2_ Hb tetramer (Storz et al., 2010; Natarajan et al., 2015a, 2015b, 2016, 2018; Tufts et al., 2015; Zhu et al., 2018; Signore et al., 2019). A regulatory mechanism of adaptation was also documented in the Tibetan antelope, whereby an increased Hb-O_2_ affinity is achieved *via* an Hb isoform switch (Signore and Storz, 2020). The convergent evolution of increased Hb-O_2_ affinity in high-altitude taxa highlights the importance of this phenotype in hypoxia adaptation (Storz, 2016). However, numerous exceptions have also been documented where high-altitude specialists have not evolved elevated Hb-O_2_ affinities in comparison with lowland sister taxa (Revsbech et al., 2013; Janecka et al., 2015).

Although an elevated Hb-O_2_ affinity can improve arterial O_2_ saturation under conditions of severe hypoxia, the resultant increase in arterial O_2_ content only translates into an increased capacity for O_2_ transport to tissues if it is associated with a sufficiently high tissue O_2_ diffusion capacity (Wearing et al., 2021). Accordingly, bar-headed geese and high-altitude deer mice have evolved elevated Hb-O_2_ affinities (Storz et al., 2010; Jendroszek et al., 2018; Natarajan et al., 2018) in conjunction with derived muscle phenotypes that improve O_2_ diffusion capacity and O_2_ utilization, as characterized by increased capillary density, oxidative fiber density, mitochondrial volume density, and mitochondrial oxidative capacity (Scott GR. et al., 2015, Scott et al., 2015 G. R.; Lui et al., 2015; Mahalingam et al., 2017, 2020; Tate et al., 2017, 2020; Nikel et al., 2018; Storz et al., 2019). High-altitude deer mice can also achieve higher cardiac output in hypoxia than low-altitude mice (Tate et al., 2020; Wearing et al., 2022). The combined effects of increases in arterial O_2_ saturation, cardiac output, and tissue O_2_ extraction lead to pronounced increases in aerobic capacity in hypoxia (Tate et al., 2020). Storz and Scott (2019) provide an overview of O_2_-transport pathway mechanisms.

**FIGURE 1:**
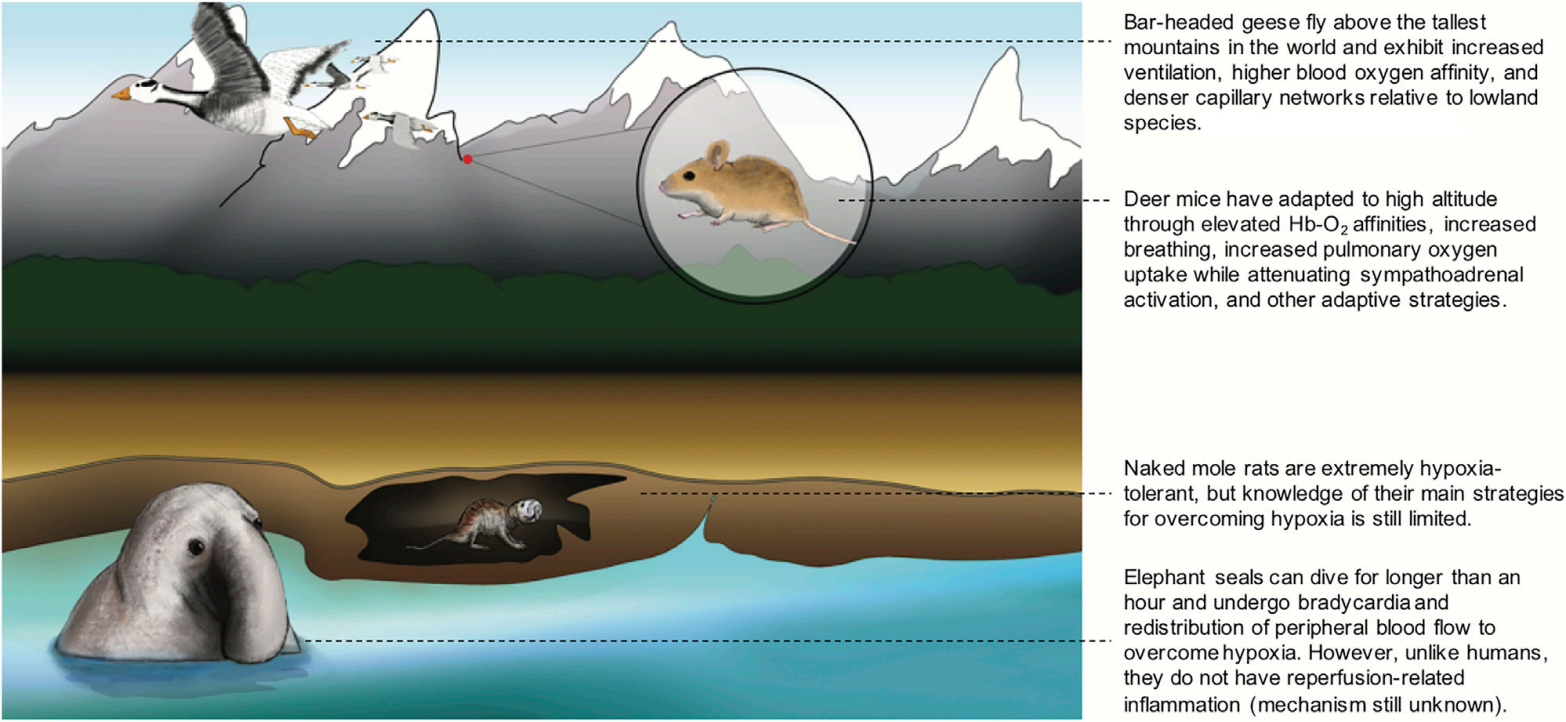
Different strategies that animals utilize to overcome hypoxia. Bar-headed geese fly above the tallest mountains in the world and exhibit increased ventilation, higher blood oxygen affinity, and denser capillary networks relative to lowland species among other adaptive traits. Deer mice have adapted to high altitude through elevated Hb-O_2_ affinities, increased breathing, increased pulmonary oxygen uptake while attenuating sympathoadrenal activation, and other adaptive strategies. Naked mole rats are extremely hypoxia-tolerant, but knowledge of their main strategies for overcoming hypoxia is still limite. Elephant seals can dive for longer than an hour and undergo bradycardia and redistribution of peripheral blood flow to overcome hypoxia. However, unlike humans, they do not have reperfusion-related inflammation (mechanism still unknown).

The distinct responses to hypoxia across high-altitude birds shows how attempts to interpret how species match O_2_ supply to O_2_ demand requires consideration of the integrated function of all the steps in the O_2_ transport cascade. Thus, while bar-headed geese demonstrate surprisingly large increases in ventilation accompanied by a fall in pulmonary O_2_ extraction in hypoxia (Scott and Milsom, 2007; Lague et al., 2016), this produces a respiratory alkalosis that should enhance Hb-O_2_ binding, increasing arterial O_2_ content (CaO_2_) and reducing the need to increase cardiac output (Lague et al., 2017). On the other hand, the high mass-specific cardiac output seen in high-altitude speckled teal and ruddy ducks despite a large O_2_ carrying capacity of the blood appears to be essential to support the high mass-specific metabolic rates of these smaller species (Ivy et al., 2019; Laguë et al., 2020; Milsom et al., 2021). In short, there is tremendous diversity in how different high-altitude avian species match O_2_ supply to demand. Different strategies animals utilize to mitigate impacts of hypoxia are illustrated in Figure 1.

### 2.3 Physiological Adaptations to Hypoxia in Marine Mammals

Some of the most dramatic mammalian adaptations to hypoxia have evolved in diving mammals that recolonized marine environments 50–30 million years ago (Berta, 2015). Unlike high-altitude or burrowing animals, marine mammals do not face issues with pulmonary-O_2_ loading since O_2_ uptake occurs at sea level. Instead, hypoxemia is induced by breath-hold diving and results from depletion of blood and muscle O_2_ stores (Thewissen et al., 2009). In deep-diving mammals, the lungs contribute very little to O_2_ storage capacity, as the animals routinely experience alveolar collapse at depth, forcing reliance on blood and muscle O_2_ stores during dives (McDonald and Ponganis, 2012). For this reason, deep-diving mammals evolved to have extremely high concentrations of Hb in blood and myoglobin in muscle as a mechanism to increase O_2_ storage capacity (Ponganis, 2015). To maximize the use of on-board O_2_ stores, marine mammals exhibit dramatic cardiovascular adjustments while diving and surfacing, known as the dive response. At the onset of a breath-hold dive, marine mammals experience dramatic reductions in heart rate, which is matched by a high degree of peripheral vasoconstriction to maintain a steady mean arterial pressure (Davis and Williams, 2012). This bradycardia and tissue ischemia ensures that hypoxia-sensitive organs, such as the brain and heart, have consistent supplies of blood arriving throughout the dive. Other organs (e.g., kidney, liver, spleen), which experience severely reduced blood delivery during dives, must utilize alternative O_2_ stores (e.g., myoglobin), or rely on anaerobic metabolism to meet energetic demands (Kooyman et al., 2021). When an animal surfaces from a dive, ischemic tissues undergo reperfusion and heart rate increases well above resting levels to maintain mean arterial pressure. Despite a lifestyle of chronic ischemia-reperfusion events to many of their tissues, these mammals appear to have no detrimental side effects and, therefore, represent excellent mammalian models to study potential cytoprotective mechanisms.

Hypoxia tolerance in marine mammals has been studied extensively in the northern elephant seal, an elite diver that spends up to eight months continuously foraging at sea and conducting dives that may be in excess of one and a half hours (Hassrick et al., 2010). Elephant seals undergo routine apneas even when on land when asleep, exhibiting physiological adjustments similar to those during dives mentioned previously (Ponganis et al., 2006; Stockard et al., 2007; Tift et al., 2013). During these terrestrial apneas, elephant seals experience a reduced muscle blood flow of only 46% of the average muscle blood flow during normal breathing (Ponganis et al., 2008). The animals routinely experience hypoxemia during these terrestrial apneas (PaO_2_ ∼ 30 mmHg), and the degree of blood O_2_ depletion during dives is even more dramatic (PaO_2_ ∼ 10 mmHg) (Meir et al., 2009). Considering these animals spend over 80% of their lives in a breath-hold, it is remarkable that their tissues can tolerate these degrees of hypoxemia and repeated ischemia/reperfusion events.

In fact, unlike humans and most other mammals, marine mammals do not show evidence of tissue damage in response to repeated bouts of severe hypoxia or ischemia-reperfusion events (Vázquez-Medina et al., 2011; Vázquez-Medina et al., 2012). Potential strategies to avoid injury include mechanisms to cope with ischemic inflammation and reperfusion-derived oxidant generation. In particular, the glutathione system likely plays a key role in antioxidant defense in marine mammals. Glutathione levels in tissues and circulation are significantly higher in diving than in non-diving mammals (Vázquez-Medina et al., 2007; García-Castañeda et al., 2017). Similarly, several genes involved in the glutathione system are under positive selection or expanded in marine mammals (Yim et al., 2014; Zhou et al., 2018).

Limiting ischemic inflammation is crucial to avoid reperfusion injury. Evidence shows that deep-diving Weddell and elephant seals possess a yet-to-be-identified anti-inflammatory component in their plasma (Bagchi et al., 2018). These same two species also have the highest levels of endogenous carbon monoxide (CO) ever measured, with levels in the blood approaching those seen in chronic cigarette smokers (Pugh, 1959; Tift et al., 2014; Tift and Ponganis, 2019). Interestingly, exposure to low or moderate levels of CO has been shown to have potent cytoprotective effects (Motterlini and Otterbein, 2010). The primary source of endogenous CO production in mammals is the degradation of heme by heme oxygenase enzymes (Tenhunen et al., 1968). Therefore, elevated Hb and myoglobin stores result in more heme that could be degraded to produce CO endogenously. The mechanism of cytoprotection is also not completely understood, but there are several studies that have now shown CO can provide tissue-specific protection against injuries associated with hypoxia and ischemia-reperfusion events (Tift et al., 2020). These unanswered questions may be best examined in the elephant seal due to its propensity for voluntary breath-holds and the existence of established endocrine, biochemical, and molecular techniques to work with the animals (Khudyakov et al., 2015; Crocker et al., 2016). Ongoing investigations using *ex vivo* systems that are amenable to physiological manipulation and molecular perturbation can also complement *in vivo* studies while providing insights into mechanisms that confer natural tolerance to hypoxemia and ischemia/reperfusion in diving mammals as described by Allen and Vazquez-Medina (2019) and Lam et al. (2020).

### 2.4 Genomic Analyses Across Multiple Species Reveal Converging Patterns of Hypoxia Adaptation

For species that have adapted to hypoxic environments, key traits show consistent genetic evidence for convergent evolution. The HIF pathway is reported as a major genetic target of selection in multiple species native to the Tibetan Plateau. For example, *Endothelial PAS Domain Protein 1* (*EPAS1*), the gene that encodes the HIF-2α subunit, is reported to be important for high-altitude adaptation in Tibetan yak and antelope (Wang et al., 2015), snakes (Li et al., 2018), dogs (Wang et al., 2014), and wolves (vonHoldt et al., 2017) (reviewed in Pamenter et al., 2020). These key adaptations in HIF-pathway genes have been well summarized recently across many hypoxia-adapted species (Storz, 2021; Storz and Cheviron, 2021), including domesticated animals (Witt and Huerta-Sánchez, 2019). While the phenotypic effects of many of these naturally occurring *EPAS1* variants are not well understood, recent studies of highland deer mice have shown that allelic variation is associated with altered cardiovascular function and transcriptomic responses to hypoxia (Schweizer et al., 2019). Specifically, the allele that predominates at high altitude is associated with an elevated heart rate under hypoxia and reduced expression of genes involved in catecholamine biosynthesis and secretion. Many of these effects seem to be attributable to a single non-synonymous substitution that disrupts the interaction between HIF-2 alpha and its transcriptional co-activator, CREB-binding protein (Song et al., 2021). Further evidence for convergent adaptation is reported at the Hb gene region across various species and impacts Hb-O_2_ affinity, as mentioned in Section 2.2 (Storz 2019).

Experiments in the fruit fly, *Drosophila melanogaster*, have also yielded insights into key genetic pathways for hypoxia adaptation, primarily involving the Notch pathway. The Notch signaling pathway is highly conserved across animal species and regulates many aspects of development, cell-cell signaling, and tissue renewal (Kopan and Ilagan, 2009). In hypoxia, Notch-responsive promoters are activated, and the HIF-1 transcription factor is recruited to these promoter sites (Gustafsson et al., 2005). Notch pathway genes were shown to underlie hypoxia adaptation and increased Notch activity conferred improved hypoxia tolerance in *Drosophila* after hypoxia exposure for 200 generations (Zhou et al., 2011, 2021) and have been reported as putatively adaptive genes in Andean populations at high altitude (Bigham et al., 2009). Research aimed at analyzing the cross-talk between HIF and NOTCH pathways in highland populations will provide greater insight into long-term hypoxia adaptations in humans (O’Brien et al., 2022).

Information from extinct species provide additional insight into the genetic factors underlying long-term hypoxia adaptation. The growing accessibility of ancient DNA sequencing coupled with *in vitro* expression systems permits the resurrection of phenotypes once lost to evolutionary time (Campbell et al., 2010; Mirceta et al., 2013; Huerta-Sánchez et al., 2014; Campbell and Hofreiter, 2015; Signore, 2016). For example, the extinct great auk (*Pinguinus impennis*) is an ideal candidate to study phenotypic changes associated with breath-hold hypoxia, as this alcid very recently diverged from its aerial relatives (razorbills and murres) and became a flightless diving specialist. While some morphological changes associated with the great auk’s air-to-sea transition are present in the fossil record, the critical physiological processes that accompanied this transition have been lost. However, ancient DNA sequencing and *in vitro* expression of the great auk’s Hb proteins suggest this species also evolved an adaptive increase in Hb-O_2_ binding affinity (Berenbrink et al., 2017). Information on ancient adaptive trends in DNA can also be gleaned from existing animals’ genomes. For example, an adaptive gene region with linked genetic makers (i.e., a haplotype) containing *EPAS1* and *Protein Kinase C Epsilon* (*PRKCE*) is thought to have originated in high-altitude Tibetan wolves and mixed into highland dogs’ genomes, a process called introgression, more than 10,000 years ago (vonHoldt et al., 2017). This introgression mirrors the finding of Denisovan (archaic human) DNA in modern Tibetan human genomes, also in the form of a haplotype containing *EPAS1* (Huerta-Sánchez et al., 2014; Hu et al., 2017). Understanding adaptive introgression in other populations and at other genomic locations could provide important insight into evolutionary processes in hypoxia-adapted species.

## 3 Time Domain 2: Adaptation to Hypoxia in Humans

### 3.1 Physiological Adaptations to Hypoxia in Humans

Compared to other high-altitude species, humans have persisted at high altitude for a relatively short period of time. Humans first inhabited high altitudes hundreds of generations ago, with reports suggesting as long as 30,000 to 40,000 years on the Tibetan Plateau (Rademaker et al., 2014; Capriles et al., 2016; Zhang et al., 2018). Although human occupation of high altitude is significantly shorter than other species mentioned above, humans display distinct physiological adaptations to hypoxia that have developed over thousands of years, and these patterns of adaptation vary by continental group (Beall, 2007; Bigham et al., 2009; Simonson, 2015; Luks et al., 2021).

While there is considerable variation within populations, studies of high-altitude residents indicate many individuals of Tibetan ancestry exhibit larger lung volumes, elevated resting ventilation, and elevated hypoxic ventilatory responses compared to both Han Chinese high-altitude residents and Andean high-altitude groups (Hackett et al., 1980; Sun et al., 1990; Droma et al., 1991; Zhuang et al., 1993; Beall et al., 1997; Curran et al., 1997; Moore, 2000; Wu and Kayser, 2006; Beall, 2007). Contrary to Tibetans, many Andean groups exhibit blunted ventilatory responses to hypoxia (Beall, 2007; Heinrich et al., 2020) with associations noted between hematocrit and daytime and sleep oxygen saturation in Andean men and women as well as blunted heart rate response to hypoxia in men (Heinrich et al., 2020).

Variation in hemoglobin concentration ([Hb]) has been well characterized and replicated in many studies of high-altitude populations. Tibetan highlanders generally maintain [Hb] with ranges typically observed in populations living at or near sea-level, while Andean highlanders have, on average, much higher [Hb] (Beall, 2007). Native Amhara high-altitude residents of the Simien Plateau of Ethiopia demonstrate similar [Hb] and erythropoietin concentrations to Tibetans and are also able to maintain higher O_2_ saturation levels than either Andeans or Tibetans (Beall et al., 2002). In contrast, Native Oromo high-altitude residents of the Bale Plateau of Ethiopia have elevated hemoglobin concentration and low O_2_ saturation (Lundgrin et al., 2013).

The precise mechanisms underlying differences in [Hb] phenotypes among high-altitude human populations remains an active area of research. Studies suggest plasma volume as the key adaptive phenotype that underlies lower [Hb] in Sherpa relative to Andean males with comparable blood volumes (Stembridge et al., 2019). In Tibetan males, relatively lower [Hb] was associated with higher peak VO_2_, which was further associated with heart and muscle diffusion components of O_2_ transport (Simonson et al., 2015). It is further plausible that variation in red cell lifespan/destruction underlie within and across population variation (Tift et al., 2020). Additional genetic studies, as discussed in Section 3.2, and further physiological and functional assessments will provide much needed insight into these mechanisms.

Differences are also apparent in traits associated with blood flow and O_2_ diffusing capacity, particularly relating to the signaling molecule and vasodilator nitric oxide (NO). Tibetans display higher exhaled NO than Andeans (Beall et al., 2001), and this elevated NO is associated with enhanced pulmonary blood flow (Hoit et al., 2005) and may account for the lower pulmonary artery pressures observed in Tibetans (Groves et al., 1993; Hoit et al., 2005; Beall, 2007). Tibetans also demonstrate higher circulating NO and bioactive NO products compared to sea-level residents, with the former being associated with variants in the regulator gene of nitric oxide synthase, *GCH1* (Guo et al., 2017), and the latter with higher forearm blood flow (Erzurum et al., 2007). Some Himalayan Sherpa also demonstrate higher sublingual capillary densities and microcirculatory blood flow at high altitude (5,300 m) compared to sea-level residents (Gilbert-Kawai et al., 2017). As discussed in Section 5.3, individual variation in NO could further underlie susceptibility to high-altitude illnesses, such as high altitude pulmonary hypertension.

While distinct adaptive phenotypes are observed across high-altitude human populations, it is important to recognize that substantial within-population variation exists among each group. Furthermore, the extent to which observed trait differences reflect genetically based adaptive changes, environmentally induced effects, and/or plasticity in terms of gene-environment interactions requires additional functional investigation (Hall et al., 2020). The likely importance of gene-environment interactions in shaping phenotypes in high-altitude natives is emphasized by recent work in deer mice, which shows that high-altitude populations have evolved altered responses to chronic hypoxia for several cardiorespiratory traits (Storz and Scott, 2019).

### 3.2 Genomic Evidence for Positive Selection in Humans

**FIGURE 2:**
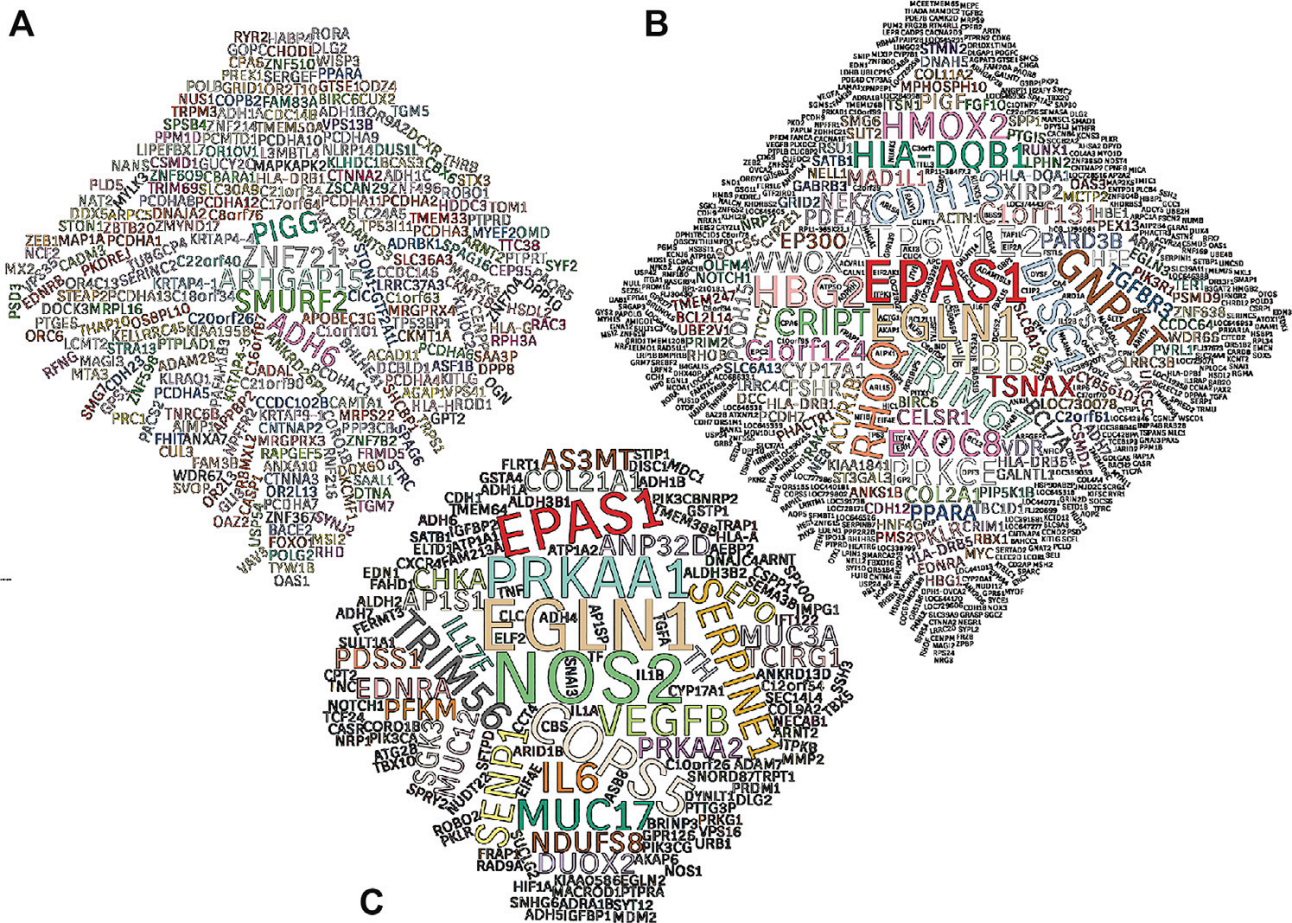
Word clouds of genes under positive selection. Genes reported as top targets of positive selection in high-altitude human populations illustrated in word clouds. A total of 31 publications were used to establish word clouds based on four, fifteen, and twelve original studies from Ethiopian **(A)**, Tibetan **(B)**, and Andean **(C)** populations, respectively. All genes included are mentioned by name in the main text of at least one study and/or mentioned more than once in the supplementary materials section of the publication. Text size is indicative of the number of times a top selection candidate gene is mentioned. Gene symbols are validated using the Ensembl genome database. Major statistical methods considered in this analysis are FST, PBS, iHS and XP-EHH. Gene lists of this analysis can be found in the Supplementary Table.

All studies reporting genetic adaptation to high altitude employ statistical tests that derive from observations that a) only two of the four forces driving genetic evolution–gene flow and natural selection–have directional effects, and b) of these, gene flow affects all loci (particular positions in the genome) in the same way, whereas natural selection acts on specific loci (Lewontin and Krakauer, 1973). While such tests have their limitations (Jensen et al., 2016), the wide range of tests employed and consistent results across multiple studies provide substantial evidence that natural selection has operated on specific genes in the multigenerational residents of Ethiopian, Himalayan, and Andean high-altitude populations. The genes reported in many of these studies are contained within regions of the genome that exhibit a distinct pattern (i.e., a “selective sweep,” summarized in Simonson (2015)). In most cases, the specific genetic change in the DNA underlying the adaptation is unknown but is likely within or near the gene reported. A compilation of genes reported in human high-altitude adaptation studies are visually summarized in Figure 2, highlighting top adaptive genes reported among Ethiopians (Figure 2A), Tibetans (Figure 2B), and Andeans (Figure 2C).

While many HIF pathway genes are reported as targets of selection in highland populations, only some are associated with known, putatively adaptive phenotypes and these associations vary across populations. In genomic studies of Tibetans, allelic variants at HIF genes such as *EPAS1* and *Egl-9 Family Hypoxia Inducible Factor 1* (*EGLN1*) exhibit significant associations with [Hb] (Beall et al., 2002; Simonson et al., 2010; Yi et al., 2010) but do not provide information about the direct modulation of this phenotype (reviewed in Simonson (2015)). Lower [Hb] in Tibetan males was also associated with regulatory variants in the *Heme oxygenase 2* (*HMOX2*) gene, reported as a top selection candidate gene in Tibetans (Simonson et al., 2010), and these regulatory variants were associated with higher *HMOX2* expression in cell culture analyses (Yang et al., 2016). These findings suggest upregulation of the heme oxygenase/carbon monoxide (HO/CO) pathway that is involved in heme degradation–the same HO/CO pathway relevant to elephant seal adaptation (discussed in Section 2.3). Elevated [Hb] in Andeans is associated with increased endogenous CO, suggesting potential convergence of elevated CO through different rates of erythrocyte production versus lifespan/destruction in each population (Tift et al., 2020).Variants within the HIF genes and other adaptive genetic factors may affect various steps in the O_2_ transport pathways and/or metabolic O_2_ utilization that prevent cellular O_2_ levels from declining as much as they would otherwise thereby attenuating the hypoxic stimulus that induces erythropoiesis or plasma volume contraction (Storz, 2021; Storz and Cheviron, 2021). The relatively low [Hb] observed in many Tibetan highlanders may potentially be an indirect consequence of how allelic variants of genes like *EPAS1* modulate components of O_2_-sensing/transport.

From an evolutionary perspective, identifying functional links between genotypes showing evidence of positive selection and reproductive success is of particular importance. One such strategy has focused on determinants of birth weight since a) birth weight is the most important determinant of neonatal or infant mortality, b) mortality risk during pregnancy and through the first year of life is greater than at any other time prior to the end of the reproductive period (i.e., the period during which natural selection is acting), and c) the profound effect of high altitude to reduce birth weight (Jensen and Moore, 1997). Tibetan and Andean ancestry confer protection against hypoxia-associated reductions in birth weight compared to newcomer groups residing at the same altitude, reviewed in Moore (2021). In Andeans, this effect appears to stem, in part, from greater uteroplacental blood flow and O_2_ delivery during pregnancy at high altitude in women of Andean versus European ancestry (Julian et al., 2009). Such effects appear to be genetic, not developmental, in origin (Julian et al., 2011). One genomic region near adaptive protein kinase, AMP-activated, alpha 1 (*PRKAA1*), the gene encoding the α1 catalytic subunit of adenosine monophosphate kinase (AMPK), not only shows evidence of positive selection in Andeans but has also been associated with greater birth weight and uterine artery diameter at high altitude, a major determinant of uteroplacental blood flow (Bigham et al., 2014). Moreover, AMPK activation *in vitro* in pregnant human uterine vessels or *in vivo* in murine models have potent vasodilator effects that are associated with maintenance of fetal growth during high-altitude exposure (Lane et al., 2020; Lorca et al., 2020). While much remains to be learned, given that drugs such as metformin activate AMPK and have been safely used in pregnancy, such studies offer the possibility of yielding new therapies for treating or preventing pregnancy disorders characterized by uteroplacental ischemia and hypoxia.

### 3.3 Evolutionary Significance in Medicine

In the fields of precision and personalized medicine, it is necessary to consider individual genetic factors that may underlie variation in particular phenotypes or pathologies. In present-day highland populations, unique evolutionary histories help contextualize such variation, i.e., adaptations and maladaptations. Each population’s genetic landscape has been shaped by standing (existing) genetic variation, admixture (mixture of genetic material from different populations), and/or *de novo* (novel) mutations that have occurred throughout hundreds of generations at high altitude. An example pertinent to the history of Tibetan adaptation is the introgression of the previously mentioned *EPAS1* gene region as mentioned in Section 2.4. The genetic sequence in this region has proven crucial for Tibetan adaptation and is more similar to an archaic human population that no longer exists, the Denisovans. The sequence of DNA at the *EPAS1* region is more similar to Denisovan DNA than other available human genome sequences, reflecting adaptive introgression from this population tens of thousands of years ago (Huerta-Sánchez et al., 2014; Hu et al., 2017).

Furthermore, while a vast majority of genome-based association studies have focused on populations of European ancestry (Sirugo et al., 2019), an understanding of physiological and genomic variation in other historically understudied populations are sorely needed to provide a more complete understanding of human variation relevant to hypoxia tolerance and genomic medicine. It is further important that as raw genomic data continue to increase in value as a global commodity, researchers ensure genome donors have a say in how their information is utilized and that part of the benefits received are returned to the original participants (Fox, 2020; Hall et al., 2020).

## 4 Time Domain 3: Physiological and Epigenetic Changes in Response to Hypoxia Within a Lifetime

In addition to long-term generational population-level adaptation, responses to acute (minutes) or chronic hypoxic stress (days) may occur in order to maintain oxygen homeostasis. These responses may be under genetic/epigenetic control and reflect ranges of species, population, and individual variation. Such differences could prove adaptive to a point or lead to maladaptive phenotypes, e.g., in cases of overcompensation such as excessive erythrocytosis (Hancco et al., 2020) as discussed in Section 5.2. The first physiological response to an acute hypoxic stimulus is the hypoxic ventilatory response (HVR), which involves an increase in minute ventilation due to the activation of the peripheral chemoreceptor mainly located in the carotid bodies (Powell et al., 1998; Teppema and Dahan, 2010; Ivy and Scott, 2015; Pamenter and Powell, 2016). This response shapes how one acclimatizes to hypoxia and has the potential to impact the extent of hypoxia experienced, thereby impacting molecular changes that may lead to a cascade of physiological changes. Epigenetic modifications, in interaction with the genome, the environment, and other regulatory factors, provide a mechanism for environmental stresses such as hypoxia to modify gene expression early in development and throughout the life course. While various steps of O_2_ transport may be altered over shorter time domains within the lifetime, this Section focuses primarily on the initial hypoxia responses involving the first steps of O_2_ transport.

### 4.1 Hypoxia Responses in Early Life Stages

The magnitude of hypoxia responses vary across life stages. The initial increase of ventilation in neonates is moderate compared to the HVR observed in adults (Teppema and Dahan, 2010; Dzal et al., 2020), and the HVR is usually accompanied by a decrease in metabolic rate not observed in adulthood (Mortola, 2004; Mortola and Maskrey, 2011). The HVR in newborns is mediated by the peripheral chemoreceptors and progressively increases with age as the result of chemosensory reflex maturation (Teppema and Dahan, 2010; Dzal et al., 2020) and a decrease of HVD with maturation (Bissonnette, 2000; Renolleau et al., 2001). The newborn phase is critical for the development of respiratory control (Carroll, 2003), as changes in O_2_ levels, such as hypoxia or hyperoxia, may cause alterations in respiratory control with long-lasting effects (Carroll, 2003; Bavis, 2005, 2020; Lofaso et al., 2007; Teppema and Dahan, 2010). Given the long-lasting repercussions of O_2_−related challenges, it is plausible that hypoxic events, *via* epigenetic modifications, may result in phenotypic plasticity. Such modifications may lead to notable physiological changes and/or distinct gene expression, proteomic, and metabolomic profiles as discussed further in this Section and the final Section of this review. Indeed, recent reports describe epigenetic changes with hypoxia exposure in neonates (Bustelo et al., 2020; Tong et al., 2021), and epigenetic modifications in peripheral chemoreceptors have been induced with hypoxia exposure (Nanduri et al., 2012, 2017a, 2017b; Prabhakar, 2013). These findings highlight the crucial impact of O_2_ levels in early stages.

### 4.2 Ventilatory Responses to Acute and Sustained Hypoxia

Ventilatory responses vary based on the duration of hypoxia exposure. The HVR is a reflex response initiated when glomus or type 1 cells in the carotid body detect a decrease in the arterial levels of oxygen, producing an increase in intracellular Ca^2+^ and release of one (or more) neurotransmitters to terminals of the carotid sinus nerve (Teppema and Dahan, 2010; Ortega-Sáenz et al., 2013; Prabhakar and Semenza, 2015; Iturriaga et al., 2021). These neurotransmitters produce an increase in the frequency of action potentials through the glossopharyngeal nerve to respiratory centers of the brainstem, resulting in activation of the phrenic nerve, activation of the diaphragm, and increased respiratory frequency and tidal volume to produce increased ventilation (Teppema and Dahan, 2010; Pamenter and Powell, 2016; Ortega-Sáenz and López-Barneo, 2020). The specific nature of the O_2_ sensor in the glomus cells is currently debated in terms of: a) metabolism-related mechanisms (i.e., processes occurring in the mitochondria), b) membrane-linked O_2_ sensor mechanisms (e.g., potassium and/or calcium channels, or olfactory receptors), and c) mechanisms involving gasotransmitters determining or modulating the chemoreceptor activity (e.g., carbon monoxide, hydrogen sulfide, nitric oxide) (Prabhakar and Semenza, 2015; López-Barneo et al., 2016; Iturriaga et al., 2021), which are all plausible targets for different adaptations. For example, how the HVR is beneficial under specific durations and patterns of hypoxia, the extent of genetic/epigenetic control versus other mechanisms of physiological plasticity, and links to reproductive outcomes at altitude are active areas of research.

### 4.3 Acclimatization

Whereas acute hypoxia induces HVR, longer exposure to a continuous hypoxic stimulus for days, weeks, or years (or chronic hypoxia) produces ventilatory acclimatization to hypoxia, which is a further increase of ventilation during hypoxic stimulation and when breathing normoxic air (Powell et al., 1998; Dempsey et al., 2014). For example, upon sojourn to high altitude, the HVR increases over 2–14 days and remains elevated, along with resting ventilation and arterial O_2_ saturation, for at least eight weeks as a manifestation of ventilatory acclimatization to hypoxia (Sato et al., 1994; Hupperets et al., 2004). While the timescale varies, and it has been shown to take over 10 days for ventilation to stabilize in some individuals (Powell et al., 1998). It has been suggested that a blunting of the HVR (hypoxic desensitization) reported among Andean populations (Chiodi, 1957; Severinghaus et al., 1966; Weil et al., 1971; Beall et al., 1997; Léon-Velarde et al., 2003) suggests an elevated ventilatory response cannot be maintained over longer time periods and will eventually decline (Zhuang et al., 1993). This idea was supported by another long-term acclimatization study showing HVR declined in some individuals of European ancestry after 45 days (Forster et al., 1971). If blunting of chemoreflex responses is typical in some sea-level residents after long-term altitude exposure and in Andean highlanders, it is intriguing that high HVR is maintained in some Tibetan highlanders. Selective pressure acting on standing and/or adaptive genetic variation or epigenetic differences in Tibetan populations may have contributed to the maintenance of this trait (Simonson et al., 2010; Simonson, 2015). Given that HVR magnitude also varies both within and across human populations, it is key genetic factors likely contribute, at least in part, to differences in ventilatory control (Collins et al., 1978; Brutsaert et al., 2005). The extent of variability in short- and long-term acclimatization responses across individuals and within various populations remains to be fully explored and would benefit from longitudinal analyses.

While the genetic underpinnings of the magnitude of HVR are not well understood, HIF regulators may play a key role in ventilatory acclimatization to hypoxia. For example, heterozygous *Hif-1α* knockout mice exposed to 72 h of hypoxia have reduced ventilation during normoxia and acute hypoxia compared to homozygous *Hif-1α* mice (Kline et al., 2002). In addition, site-specific deletion of *Hif-1α* in the brainstem nucleus tractus solitarius of adult mice did not affect the acute HVR in normoxia but blunted the acute HVR in mice exposed to chronic hypoxia for 7 days (Moya et al., 2020). Hodson et al. (2016) showed that inducible inactivation of the HIF regulator prolyl hydroxylase 2 (Phd2) in mice resulted in an increased HVR, and that deletion of *Epas1*, which encodes for Hif-2α, but not Hif-1α, prevented this increase of HVR. Additional work also suggests that *Epas1* and potentially other genetic components may play a key role in ventilation, primarily through O_2_−sensing in glomus cells of the carotid body (Bishop and Ratcliffe, 2020; Moreno-Domínguez et al., 2020). Moreno-Domínguez et al. (2020) ablated *Epas1* in mice, which resulted in a reduced HVR. They achieved a similar reduction in HVR when they genetically deleted *Cox4i2*. Studies regarding the genetics of the HVR should be a prolific area for future experimental studies.

While increases in ventilation and also heart rate are immediate responses to hypoxia, other physiological changes can occur within days and weeks to improve tissue O_2_ delivery in chronic hypoxia (Imray et al., 2011). Chronic mild hypoxia, between 8 and 12% O_2_, triggers profound vascular remodeling in the central nervous system (CNS) of adult mice, resulting in a greater than 50% increase in blood vessel density throughout the CNS over a period of 2–3 weeks (Boroujerdi and Milner, 2015). This process encompasses an angiogenic response that includes endothelial proliferation (Li et al., 2010), arteriogenic remodeling (Boroujerdi et al., 2012), enhanced transient expression of remodeling proteins such as fibronectin (Boroujerdi and Milner, 2015), and sustained elevated expression of proteins involved in blood-brain barrier integrity (e.g., tight junction proteins and vascular basement extracellular matrix proteins such as laminin) (Li et al., 2010). Such changes may provide protection in stroke and multiple sclerosis as discussed in Section 5.4.

### 4.4 Hypoxia and the Epigenome Across the Lifespan

Hypoxia in early life environments may have profound and lasting effects on the development of adult physiology through many different molecular, physiological, and morphological changes. Epigenetics is most broadly defined as the study of mitotically heritable changes to DNA that do not alter the nucleotide sequence (Holliday, 1987). The most commonly studied epigenetic mechanisms include DNA methylation (methyl groups modifying cytosines preceding guanines, or CpG sites), histone modifications (methyl, acetyl, and other chemical tags modifying the proteins around which DNA is wrapped), and non-coding RNAs (Allis and Jenuwein, 2016). Because the epigenome sits at the nexus of the genome and the environment, it is a layer of regulation that is of particular interest in the study of early hypoxia exposures.

Some regions of the epigenome are characterized by increased plasticity during the critical window in human development from preconception to early childhood (Hochberg, 2011). During this time, the epigenome undergoes active and passive reprogramming and thus is very susceptible to environmental influences (Faulk and Dolinoy, 2011; Buganim et al., 2013). Upon gamete formation, and again after fertilization, DNA methylation is erased and re-established *de novo* in a cell- and tissue-specific manner (Reik and Dean, 2001; Seisenberger et al., 2012). Moreover, mounting evidence suggests that early life events such as mother’s psychosocial stress levels, nutrition, and exposure to heavy metals and endocrine disrupters such as bisphenol-A (BPA), can affect DNA methylation, predisposing a developing child to adverse health outcomes later in life (Dolinoy et al., 2007; Senut et al., 2012; Klengel et al., 2014; Thayer and Non, 2015; Non, 2021).

The epigenetic processes involved in adaptation to high-altitude hypoxia are just beginning to be explored, as summarized by Julian (2017, 2019). Recently developed animal models have revealed how epigenetic changes contribute to negative outcomes in prenatal hypoxia exposure. For example, in rats, epigenetic changes induced by hypoxia in critical stages of prenatal development can lead to increased cardiac vulnerability to hypoxia in adults (Xiong et al., 2016) and epigenetic reprogramming in response to maternal hypoxia that manifests in adult offspring (Lv et al., 2019). Additionally, intermittent hypoxia exposure in late gestation of mice has shown to induce DNA methylation changes across nearly 700 genes, many associated with metabolic regulation and inflammation, in adult male offspring (Khalyfa et al., 2017).

While early life is hypothesized to be a sensitive window for epigenetic changes, exposure to hypoxia across the lifespan can also result in epigenetic changes. Alkorta-Aranburu et al. (2012) analyzed epigenetic differences across adult human populations living at different altitudes in Ethiopia and identified four CpG sites with significantly different methylation levels in saliva samples from high- versus low-altitude Oromo Ethiopians. However, these sites were not found in genes known to be relevant to hypoxia response. A large study of epigenetic variation in hundreds of Quechua individuals in Peru living at high versus low altitude identified associations with DNA methylation at two different loci (Childebayeva et al., 2019b). Specifically, time spent at high altitude was associated with higher levels of DNA methylation at the repetitive element known as the *Long Interspersed Nuclear Element 1* (*LINE-1*) throughout the genome and lower methylation levels at the promoter region of *EPAS1*. Moreover, a few epigenome-wide studies of lifetime and early developmental altitude exposures in the same population of Andeans found differentially methylated loci in various genes, including *Superoxide Dismutase 3* (*SOD3*), a gene that plays a role in antioxidant defense against oxidative stress, as well as accelerated epigenetic aging in those living at high relative to low altitude (Childebayeva et al., 2021a). The authors also identified associations between DNA methylation and the altitude adaptive phenotype of fraction of exhaled nitric oxide (Childebayeva et al., 2021a). Surprisingly, even short-term hypoxia, such as that experienced by Europeans ascending Everest, elicited distinct epigenetic changes at key HIF loci, including *EPAS1* (Childebayeva et al., 2019a), along with other genes in HIF and RAS pathways (Childebayeva et al., 2021b). These findings speak to plasticity not just in development, but potentially throughout one’s lifetime, suggesting epigenetic factors may play a role in acclimitization to high altitude. Other mechanisms of developmental physiology at high altitude are discussed elsewhere (Jochmans-Lemonie and Joseph 2018), including changes to the cardiorespiratory system, as well as thermoregulatory processes during post-natal development.

Experimental studies in animal models also yield important insights into epigenetic modulation in the context of hypoxia. Adult rats exposed to long-term (30 days) intermittent hypoxia had higher levels of DNA methylation and down-regulation of genes related to antioxidant enzymes, such as superoxide dismutase genes *Sod1*, *Sod2*, *catalse* (*Cat)*, *Thioredoxin reductase 2* (*Txnrd2)*, *Peroxiredoxin 4* (*Prdx4*), and *Glutathione peroxidase 2* (*Gpx2)*, measured in the carotid body, the adrenal medulla, and brainstem regions associated with the carotid body reflex (Nanduri et al., 2017a). The same methylation differences were not seen in rats exposed to short-term (10 day) intermittent hypoxia, suggesting sustained or chronic exposure is necessary to produce these epigenetic effects. Of note, the carotid body has a distinct response to intermittent versus chronic hypoxia, which may explain these differences (Nanduri et al., 2017a). Furthermore, treatment with a DNA methylation inhibitor during hypoxia exposure or recovery blocked epigenetic changes and led to less reactive O_2_ species and stabilized blood pressure and breathing, suggesting that epigenetics play a role in the pathology of long-term intermittent hypoxia exposure through regulation of antioxidant enzymes (Nanduri et al., 2017a).

## 5 Time Domain 4: Hypoxia and Disease Throughout the Life Course

### 5.1 Hypoxia and Sleep

Sleep is a distinct state from wakefulness and a uniquely susceptible window for hypoxic exposures. After sleep onset, compensatory ventilatory responses to gas exchange abnormalities and upper airway obstruction can influence oxygenation patterns (Khoo et al., 1996; Wellman et al., 2011). If ventilation remains steady and sleep remains continuous, sustained hypoxia ensues. In contrast, vigorous ventilatory response and arousals from sleep result in transient increases in ventilation and oxygenation, resulting in intermittent hypoxia. Thus, individual differences in ventilatory reflexes and co-morbid cardiopulmonary disease give rise to unique patterns of hypoxia, which vary in frequency and severity.

Hypoxia is a cardinal feature of sleep-disordered breathing and has been implicated in the pathogenesis of cardiovascular and metabolic comorbidities (Drager et al., 2015). Obstructive sleep apnea (OSA) is the most common form of sleep disordered breathing and affects up to one billion people worldwide (Benjafield et al., 2019). OSA is characterized by repetitive pharyngeal collapse during sleep, leading to intermittent hypoxemia and hypercapnia with associated catecholamine surges and arousals from sleep (Dempsey et al., 2010). OSA has been associated with neurocognitive, cardiovascular, and metabolic sequelae including pulmonary hypertension (Sajkov et al., 1999). Therapy with continuous positive airway pressure (CPAP) alleviates hypoxemia, reduces systemic blood pressure, improves neurocognitive performance (Muñoz et al., 2000; Dempsey et al., 2010), and normalizes pulmonary artery pressures (Sajkov et al., 2002). CPAP may also reduce cardiovascular risk (Sassani et al., 2004; Buchner et al., 2007; Kohler et al., 2008), although data are still evolving (McEvoy et al., 2016). The causal pathway describing the role of OSA-induced cardiovascular comorbidities remain under investigation. Some of those mechanisms include carotid body activation, epigenetic changes, hypercapnia, autonomic function, inflammatory pathways, and oxidative stress (Dempsey et al., 2010; Mesarwi et al., 2015; Iturriaga, 2018; Benjafield et al., 2019).

Animal models have long been used to investigate the role of hypoxia in the pathogenesis of sleep-disordered breathing-related cardiometabolic disease. In seminal work, Fletcher et al. (1992) illustrated that chronic intermittent hypoxia (CIH) mimicking severe OSA caused an increase in systemic blood pressure. Since that time, several other investigators have extended these findings and showed that IH, during daylight hours when rodents are normally asleep, leads to dysglycemia and insulin resistance (Polotsky et al., 2003; Iiyori et al., 2007), dyslipidemia (Li et al., 2005), atherosclerosis (Savransky et al., 2007), and pulmonary hypertension (Fagan, 2001; Campen et al., 2005), supporting concurrent findings in human subjects. Studies in mice exposed to overlap (sustained and intermittent) hypoxia revealed this combined stress leads to systemic and pulmonary hypertension without protective effects typically associated with sustained hypoxia (Zhen et al., 2021). We have derived considerable mechanistic insight about the role of hypoxia in sleep and pulmonary illness from these and other such studies.

In addition to OSA, central sleep apnea (CSA) is common in high-altitude sojourners. During acute high-altitude exposure, the increase in ventilatory chemosensitivity, particularly to hypoxia, leads to unstable breathing patterns (Burgess and Ainslie, 2016). Desaturation events cause periods of hyperventilation which result in hypocapnia-induced apneas or hypopneas, which lead to subsequent desaturation events and arousals. The severity of this periodic breathing pattern appears to increase at higher elevation and may worsen or improve with acclimatization depending on the elevation and degree of increase in the hypoxic ventilatory response (Andrews et al., 2012; Burgess et al., 2013; Orr et al., 2018; Frost et al., 2021). In long term high-altitude residents, sleep disordered breathing remains common and may be linked to the development of excessive erythrocytosis or chronic mountain sickness as discussed in the next Section. Several studies report more frequent central and/or obstructive apnea events in individuals with chronic mountain sickness compared to individuals without chronic mountain sickness at the same elevation (Sun et al., 1996; Julian et al., 2013; Rattner et al., 2014; Pham et al., 2017; Heinrich et al., 2020). Continuous desaturation events on top of existing chronic hypoxemia may lead to significant oxidative stress and inflammatory events which could play key roles in this pathogenesis.

### 5.2 Chronic Mountain Sickness

Chronic Mountain Sickness (CMS) is a manifestation of maladaptation to life at high altitude and affects a large number of people living above 2500 m (Villafuerte and Corante, 2016). The excessive production of red blood cells (excessive erythrocytosis) characterizes the condition and is associated with signs and symptoms that affect an individual’s well-being, social life, and frequently employment (Villafuerte and Corante, 2016). While increased erythrocytosis improves oxygen content, excessive levels are maladaptive. At present, it is well established that chronic hypoxemia resulting from life at high altitude is the main underlying factor; however, the main pathophysiological mechanism remains elusive. As mentioned in Section 4.3, loss of ventilatory acclimatization to altitude hypoxia leading to central hypoventilation has been proposed as the principal mechanism explaining accentuated hypoxemia and the subsequent excessive erythropoietic response (León-Velarde and Richalet, 2006). However, the significant variability in the apparent causes of excessive erythrocytosis suggests that the origin of the condition involves multiple levels (Hancco et al., 2020). Some individuals with CMS develop severe hypoxemia, possibly due to depressed ventilation during day or night that triggers excessive erythrocyte production while others have moderate hypoxemia but increased plasma erythropoietin and erythropoietin availability (as determined by the decreased soluble form of the erythropoietin receptor), or increased local erythropoietin production or sensitivity at the bone marrow level (Hancco et al., 2020). This variability suggests genetic adaptation and lack of adaptation at various levels in highlanders from the same population with and without CMS (Zhou et al., 2013).

### 5.3 High-Altitude Pulmonary Hypertension and Pulmonary Edema

**FIGURE 3:**
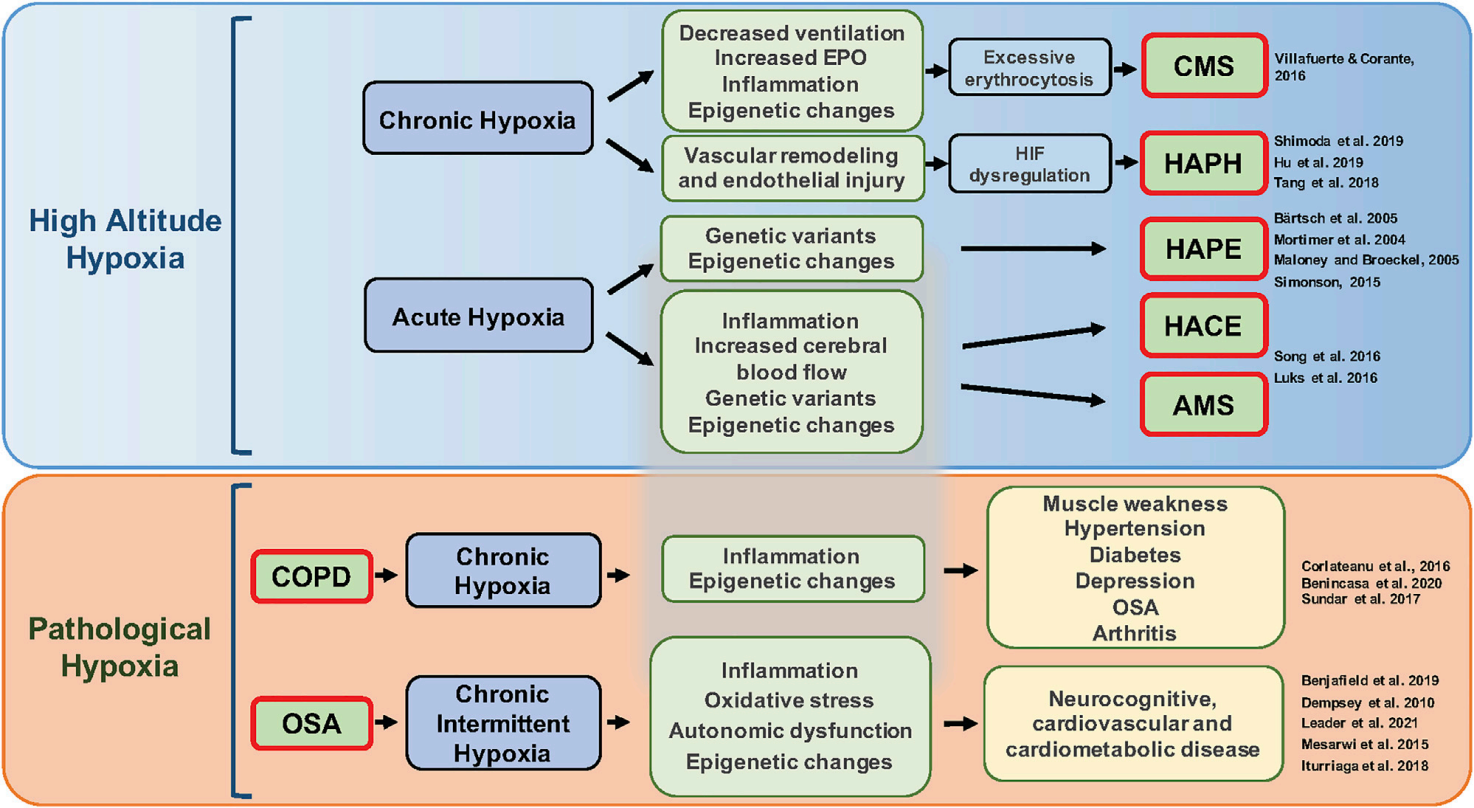
Pathological conditions associated with different patterns of hypoxia. Acute or chronic exposure to high-altitude hypoxia (upper panel) can lead to several diseases including chronic mountain sickness (CMS), pulmonary hypertension, high-altitude pulmonary edema (HAPE), high-altitude cerebral edema (HACE), and acute mountain sickness (AMS). Mechanisms underlying this variation include genetic factors, plasticity and/or lack of plasticity in ventilatory responses to hypoxia, excessive erythrocytosis, hypoxia-inducible factor (HIF) dysregulation, epigenetics, and inflammation. Additionally, clinical diseases such as chronic obstructive pulmonary disease (COPD) and obstructive sleep apnea (OSA) lead to chronic (sustained) hypoxia and chronic intermittent hypoxia, respectively (lower panel) and can trigger subsequent inflammation, oxidative stress, changes in gene expression, autonomic dysfunction, and hypercapnia that further contribute to various comorbidities.

Hypoxic pulmonary vasoconstriction (HPV) refers to the contractile response of pulmonary blood vessels in response to reduced alveolar O_2_ tension (Sylvester et al., 2012; Swenson, 2013). Contrary to what happens in systemic circulation, the local hypoxic stimulus produces HPV and reduces the blood flow to areas of the lung that are poorly ventilated. Chronic hypoxia can produce arterial remodeling and changes in vascular reactivity inducing a sustained increase in pulmonary arterial pressure leading to high-altitude pulmonary hypertension (HAPH) (Xu and Jing, 2009). These diseases and other pathological conditions associated with hypoxia are illustrated in Figure 3.

HAPH is a clinical condition characterized by high pulmonary arterial pressure or high systolic pulmonary arterial pressure, as well as right ventricular hypertrophy, heart failure, and absence of excessive erythrocytosis (León-Velarde et al., 2005). Changes in the levels and function of HIFs are involved in the mechanisms producing HAPH (Tang et al., 2018; Hu et al., 2019; Shimoda et al., 2019) but, more specifically, modulation of HPV through changes in the activity of K^+^ or Ca^2+^ channels, or vasoactive molecules such as nitric oxide or endothelin-1 (ET-1), could contribute to the development of HAPH (Sylvester et al., 2012; Mirrakhimov and Strohl, 2016). However, the fact that HAPH is not normalized by O_2_ inhalation, or agents that inhibit HPV, suggests other factors could also contribute to HAPH (Sylvester et al., 2012). HAPH is more prevalent in men than women who haven’t reached menopause (Aldashev et al., 2002, 2005) and varies among different populations living at high altitude (Fagan and Weil, 2001), which suggests a genetic component, as reviewed by Eichstaedt, Benjamin, and Grünig (2020) with potential involvement of various candidate genes (Eichstaedt et al., 2020)Aldashev et al. (2002). In support of this idea, HAPH is more common in Andean than Tibetan populations, with pulmonary arterial pressures in Tibetans living at 3,658 m comparable to sea level values (Groves et al., 1993).

Additional candidate genes that may play a role in HAPH include those involved in the nitric oxide-associated pathways due to their vasoactive properties. As mentioned in Section 3.1, exhaled nitric oxide is substantially elevated in Tibetans compared to Andeans highlanders and lowlanders at sea level (Beall, 2007). León-Velarde and Mejía (2008) suggest that variants in *ENOS* (the gene encoding endothelial nitric oxide synthase) are associated with endogenous NO production and may contribute to HAPH susceptibility (Sofowora et al., 2001), but these observations have yet to be experimentally validated. Other genes linked to nitric oxide-associated pathways are reviewed in Eichstaedt, Benjamin, and Grünig (2020). It remains difficult to dissect the direct physiopathology of HAPH from mechanisms present in common comorbidities such as high-altitude pulmonary edema and CMS.

In severe cases, HAPH can lead to high-altitude pulmonary edema (HAPE). HAPE occurs when excessive HPV leads to increased alveolar capillary permeability and fluid leak into the lung tissue. While HAPE pathophysiology is well characterized, there are limited predictors of individual susceptibility (Bärtsch et al., 2005). Individual genetic variants and epigenetic markers have been correlated with aspects of HAPE pathophysiology and susceptibility but validation of these isolated ‘hits’ is lacking (Mortimer et al., 2004; Maloney and Broeckel, 2005; Simonson, 2015). Our understanding of how genomic influences relate to short- and long-term hypoxic and hypobaric physiology is limited in part due to isolated candidate genes analyses (Simonson and Malhotra, 2020), limited sample sizes, and lack of stratification for genotype-phenotype analyses. Recently, chronic, as opposed to acute, altitude exposure has been identified as a potential cause of a type of “mountain residence” HAPE (Ebert-Santos, 2017), challenging the paradigm that all HAPE is triggered by acute altitude exposure. Thorough characterization of this chronic-induction population, in rigorous comparison with those susceptible to classic acute-induction HAPE, would be a logical first step in learning about long-term altitude exposure.

The mechanisms underlying HPV involve processes that carry over from the transition from placental-fetal oxygenation to pulmonary O_2_ supply at birth. However, these responses may not prove beneficial in severe lung disease and do not provide an advantage at high altitude. Therefore, the mechanisms essential at a crucial time point of birth has a cost under different environmental or pathological conditions later in life. While HPV contributes to complications in HAPE and OSA, it also occurs in patients with acute lung injury and pneumonia (Naeije and Brimioulle, 2001). HPV is an evolutionarily conserved response, observed in a variety of species including humans, with analogs in the gill circulation of fish and skin circulation of amphibians (Moudgil et al., 2005). HPV was first described as an adaptation by Euler and Liljestrand (1946) who recognized that HPV improves O_2_ uptake by diverting pulmonary blood flow to better aerated parts of the lungs. This physiologic effect is put to use during lung surgery when one lung is purposefully not ventilated in order to minimize intraoperative bleeding (Dunham-Snary et al., 2017). HPV compensates for regional alveolar hypoxia, as in bronchopneumonia, but not hypoxia affecting the whole lung, as in OSA and HAPE. Oral and intravenous pulmonary vasodilators, e.g., calcium channel blockers, interfere with HPV and can worsen oxygenation and mortality in critically ill lung patients (Naeije and Brimioulle, 2001; Karam et al., 2017). However, vasodilators are recommended for patients with HAPE. This difference reflects the potential beneficial role of HPV in lung injury/pneumonia and its pathological activation in HAPE. Hypobaric hypoxia is a novel condition for which lowland dwelling humans have not had time to evolve an optimal response. By contrast, some groups with a long history of high-altitude residence have a blunted HPV which might protect them against pulmonary hypertension and HAPE (Dunham-Snary et al., 2017). Other high-altitude taxa exhibit reduced HPV, such as yaks (Anand et al., 1986), llamas (Reyes et al., 2020), the Tibetan pika (Ge et al., 1998), and high-altitude deer mice (West CM. et al., 2021), providing a likely example of convergent evolution.

### 5.3 Hypoxia and Inflammatory Responses

Hypoxia may also play a key role in initiating inflammation or exacerbating preexisting inflammatory states (Eltzschig and Carmeliet, 2011; Pham et al., 2021). One example is chronic obstructive pulmonary disease (COPD), whereby chronic low-grade systemic inflammation contributes to development of comorbidities including weight loss and muscle wasting, hypertension, diabetes, depression, obstructive sleep apnea, and arthritis (Corlateanu et al., 2016). It is possible that epigenetic changes are part of the mechanisms underlying such phenotypes in COPD (Corlateanu et al., 2016; Sundar et al., 2017; Benincasa et al., 2021). The role of hypoxia and inflammatory in more acute conditions, e.g., COVID-19, remains an active area of research (Simonson et al., 2021).

Another example of hypoxia-induced inflammation is observed in some high-altitude research studies, since inflammation has been implicated as a contributing factor to acute mountain sickness (AMS), development of HAPE (Duplain et al., 2000; Hartmann et al., 2000; Grocott et al., 2007; Lemos et al., 2013; Boos et al., 2016), and high-altitude cerebral edema (HACE) (Song et al., 2016; Luks et al., 2017). Additionally, genes encoding inflammatory cytokines are found to be under evolutionary selection in Andean and Tibetan populations (Foll et al., 2014), which may afford protection instead of exacerbating hypoxemia.

Animal models indicate that inflammation also contributes to ventilatory acclimatization to hypoxia. For example, rats treated with ibuprofen during exposure to sustained hypoxia showed a blocked response to acute hypoxia without affecting the persistent hyperventilation in normoxia. Ibuprofen treatment also prevented the increase of interleukin 1β (IL-1β) and interleukin-6 (IL-6) in the nucleus tractus solitarius when compared to rats exposed to sustained hypoxia but administrated with saline (Popa et al., 2011). Stokes et al. (2017) showed that inhibition of microglia in rats using minocycline prevented the complete development of ventilatory acclimatization to hypoxia with blunted responses to acute hypoxia. In addition, their results indicate minocycline prevented the increase of IL-6 observed in the nucleus tractus solitarius of rats exposed to hypoxia per 24 h (Hocker et al., 2017).

After tissue trauma and injury, HIF-1 appears to protect the host by preventing infection (Bogdanovski et al., 2017) *via* regulating inflammation and increasing bactericidal capacity of phagocytes (Peyssonnaux et al., 2005) and further stimulates angiogenesis and promotes tissue repair (Umschweif et al., 2013). Attenuated HIF-1α signaling and impairment of downstream immune responses are theoretical concerns when supplemental O_2_ is given for patients with infection. Although impaired O_2_ delivery in sepsis and septic shock was thought to cause multiple organ dysfunction and mortality (Tuchschmidt et al., 1992), multiple trials aimed at increasing O_2_ delivery in sepsis have failed to show a benefit for this approach (Hayes et al., 1994; PRISM Investigators et al., 2017). Some studies suggest that excess O_2_ can harm sepsis patients (Demiselle et al., 2018; Perner et al., 2020). The appropriate O_2_ target in critical illness is a controversial topic that is the focus of ongoing trials (Perner et al., 2020).

### 5.4 Therapeutic Applications

Hypoxia is generally considered to impair physiological function and limit performance. However, there is mixed evidence that chronic (hypobaric) hypoxia may be beneficial in certain diseases. Some studies suggest high-altitude residents have decreased rates of cancer, e.g., lung cancer rates are lower in Peru compared to the United States after controlling for known confounding factors such as cigarette smoking (Thiersch et al., 2017; Thiersch and Swenson, 2018). Further studies are necessary to show specific effects of hypoxia, such as determining if cancer risks differ between those who reside at high altitude and those who have moved to sea level.

Chronic intermittent hypoxia (CIH), which can occur with obstructive sleep apnea, is usually considered harmful as a cause of inflammation and oxidative stress (Labarca et al., 2020), resulting in well-known pathological effects on cardiovascular physiology and metabolism (Marin et al., 2005; Barnes et al., 2022). However, CIH has also been reported to produce beneficial effects. For example, 15 cycles in inspired O_2_ administered daily to produce cycles of arterial P_O2_ similar to those occurring with OSA can improve motor neuron function in both preclinical and clinical studies (Vose et al., 2022). This line of research is based on the observation that CIH can result in long term facilitation (LTF) of phrenic nerve activity, and increased ventilation that persists in normoxia following CIH (Gonzalez-Rothi et al., 2015). Hence, it is possible that there may be some beneficial effects of hypoxia that interact with harmful effects and their complex interaction might influence evolutionary responses to hypoxia. There are several examples of such balancing selection, such as the well-known effect of sickle cell disease to provide an evolutionary advantage by protecting against malaria despite causing cardiovascular disease (Piel et al., 2010).

Hypoxic pre-conditioning at this level of hypoxia confers marked protection in several animal models of neurological disease including ischemic stroke and multiple sclerosis (Miller et al., 2001; Dore-Duffy et al., 2011; Dunn et al., 2012), raising the question of therapeutic application of hypoxic pre-conditioning as mentioned in Section 4.3. However, the optimal dose, duration and frequency of hypoxic treatment that confers maximal protection from neurological disease, and the cerebrovascular impact of “naturally occurring” hypoxia, e.g., high altitude residents, aircrew, and patients with cardiopulmonary disease, sleep apnea, and other respiratory pathologies, remains unknown.

Pharmacological interventions to increase Hb-O_2_ affinity, observed in may highland species as discussed in Section 2.2, have shown to improve hypoxia tolerance in mice, indicating this as a potential therapeutic avenue for hypoxia-induced pathologies (Dufu et al., 2017). A drug called Voxeletor (previously GBT440) increases Hb-O_2_ affinity through its interactions with hemoglobin S to inhibit polymerization and is currently being tested in sickle cell disease patients (Estepp, 2018). Several other therapies revolve around promoting increased persistence of expression of fetal hemoglobin (HbF), an isoform of Hb which is produced around 6 weeks of pregnancy (Linch, 1998) and usually persists for 2–4 months after birth (Schechter, 2008). HbF has a higher affinity to O_2_ than adult Hb, allowing for the fetus to more efficiently scavenge O_2_ *in utero* and as a neonate (Wang and Zhao, 2010). HbF is found in approximately 3–7% of adult red blood cells; however, these levels are increased in individuals with beta-thalassemia, sickle cell disease, or acute erythropoietic stress (Italia et al., 2007; Kim et al., 2015) and the extent of individual variation on a global level has yet to be established. Some individuals with sickle cell disease and HbF persistence lack the symptoms and phenotypes associated with disease, offering promising directions for therapeutic development (Fathallah and Atweh, 2006). Natural variation in a number of genes have been associated with fetal Hb persistence, with several mechanisms described (Thein et al., 2009), along with studies to develop therapies that chemically modulate HbF levels. One mechanism involves reducing the amount of adult Hb in diseases like alpha- or beta-thalassemia, resulting in observed upregulation of HbF to compensate for the lack of adult Hb genes (Wahed and Dasgupta, 2015). Other mechanisms have been attributed to mutations within the promotor regions of the genes *HBG1* and *HBG2* (Karakaş et al., 2015). These promotor variants can result in new transcription factors binding to the promotor or disruption of the binding on major repressors, such as *BCL11A* and *ZBTB7A*, allowing for continued expression of HbF (Thein et al., 2009; Martyn et al., 2018). The use of compounds such as hydroxyurea have also been shown to promote HbF expression in sickle cell disease patients (Cokic et al., 2003). Studies have tied this effect to increases in nitric oxide radical and cyclic-GMP levels (Cokic et al., 2003) while alternative factors have been proposed such as selective vasodilation or decreased platelet activation (Glover et al., 1999).

## 6 Application of Multi -Omic Tools to Advance the Field

**FIGURE 4:**
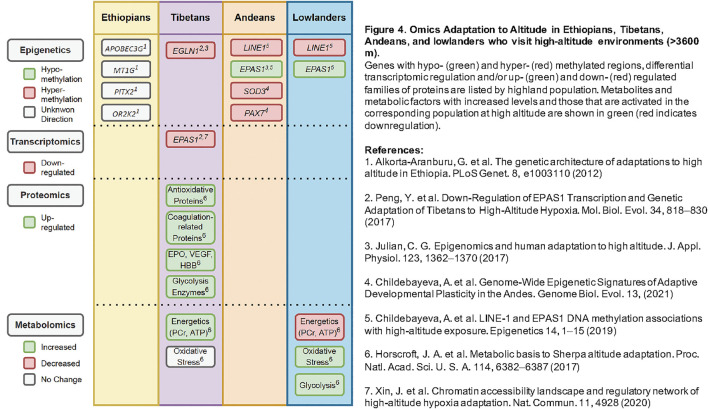
Omics Adaptation to Altitude in Ethiopians, Tibetans, Andeans, and lowlanders who visit high-altitude environments (>3600 m). Genes with hypo- (green) and hyper- (red) methylated regions, differential transcriptomic regulation and/or up- (green) and down- (red) regulated families of proteins are listed by highland population. Metabolites and metabolic factors with increased levels and those that are activated in the corresponding population at high altitude are shown in green (red indicates downregulation).

Adaptation and acclimatization to hypoxia involve coordinated efforts across many biological systems within the human body. These systems can be studied in parallel through multiple different “-omics” approaches, highlighted in Figure 4, which summarizes the various epigenetic, transcriptomic, proteomic, and metabolomic studies of high-altitude adaptation to date. As proximal phenotypes, these data may provide insight into the prioritization of precise genetic variants, which remain largely unknown, and ways to test their functional significance in highlanders and other human populations (e.g., lowland groups with shared genetic variation). With more comprehensive collections of -omics data, it is possible to generate hypotheses about mechanisms underlying hypoxia adaptation that can be tested through gene-editing and functional investigations (Hall et al., 2020).

### 6.1 High-Altitude Hypoxia Transcriptomics and Proteomics

With the advent of high-throughput sequencing and improved pipelines for sequencing technologies, information about global gene expression and regulation can now be obtained *via* RNAseq, which determines RNA quantity in a given sample (Wang et al., 2019). In a multi-omics data analysis in Tibetans that included transcriptomics analyses, Xin et al. (2020) developed a statistical model that correlated regulatory elements with gene expression, ultimately indicating down-regulation of *EPAS1* as potentially adaptive in Tibetans, corroborating previous experimental evidence from studies such as Peng et al. (2017). Findings of Xin et al. (2020), among others, demonstrate the depth and power behind the use of multiple -omics to identify potential adaptive mechanisms outside protein-coding variants in the genome. In addition to these studies in Tibetans, recent research has focused on -omics profiles in individuals acclimatizing to altitude as discussed in Section 6.2. Additional studies focused specifically on high-altitude acclimatization provide greater insight into short-term hypoxia responses that, when coupled with -omics data, yield important insight into potential mechanisms of individual hypoxia responses as well (Subudhi et al., 2014).

Proteomics is the global analysis of proteins and, in conjunction with mass spectrometry, can provide additional insight into high-altitude adaptation (Altelaar et al., 2013; Gao et al., 2017). As reviewed by Gao, Luo and Ni (2017), the majority of proteomic studies in relation to high altitude have focused on either acclimatization of lowlanders or high-altitude related illnesses. Proteomic studies on highlanders are rare, but studies such as Ahmad et al. (2013) and Du et al. (2019) have reported differentially expressed proteins, mainly part of the inflammatory pathway, involved in coagulation cascades, antioxidative stress, and glycolysis. Both studies compared plasma of native highlanders to non-acclimatized lowlanders.

In addition to studying highlanders, other proteomic studies have attempted to identify novel biomarkers for AMS (discussed in Section 5.4). For example, Julian et al. (2014) measured plasma protein levels of individuals residing in the Denver, Colorado, United States, metropolitan area (1,650 m) after acute hypobaric hypoxia exposure, comparing the protein levels of those susceptible to AMS and those resistant to AMS. They found an increase in the abundance of proteins with antioxidant properties in individuals susceptible to AMS but not in those resistant to AMS. Lu et al. (2018) conducted a similar study in individuals of Chinese ancestry and found reduction of proteins related to tricarboxylic acid cycle, glycolysis, ribosome, and proteasome in the AMS resistant (AMS−) group, but not in AMS susceptible (AMS+) group. While all the aforementioned studies analyzed plasma, Jain et al. (2018) analyzed saliva for potential proteomic biomarkers. They also found increases in levels of antioxidant enzymes in addition to several other proteins, including apoptosis inducing factor-2. Their results indicate that proteomic analysis of saliva may be a feasible, non-invasive method to measure acclimatization, or more importantly, failure to acclimatize in disease states such as HAPE or HACE.

### 6.2 High-Altitude Hypoxia Metabolomics Across Time Domains

Most adaptive traits identified in highland populations to date involve adjustments in O_2_ delivery, yet tolerance to hypoxic environments also includes adjustments in cellular O_2_ utilization and particularly to mitochondrial oxidative metabolism (Murray, 2016). One functional tool employed to investigate the complex metabolic interactions occurring in response to an environmental perturbation is metabolomics (O’Brien et al., 2015). Application of this approach in high-altitude studies has revealed metabolic signals of adaptation and acclimatization and evidence for significant remodeling of metabolism at a tissue-specific and system-wide level.

In the Himalayan Sherpa, metabolomics was employed alongside measures of mitochondrial respiratory capacity in skeletal muscle, thus combining *ex vivo* functional measures with metabolite levels *in vivo*, to assess metabolic alterations with ascent to 5300 m (Horscroft et al., 2017). Despite the fall in O_2_ delivery with ascent, Sherpas demonstrated increased skeletal muscle ATP and phosphocreatine (PCr) concentrations, suggesting improvement in energetic reserve, alongside no change in oxidative stress markers. This remarkable preservation of muscle energetics and protection against oxidative stress was accompanied by a shift away from fatty acid oxidation (FAO), with suppression of both FAO capacity and expression of transcriptional regulator of fatty acid metabolism PPARα (Horscroft et al., 2017). The O_2_ requirement of ATP synthesis is greater during FAO than glucose oxidation. This shift away from FAO therefore suggests an adaptive hypometabolic state and reduction in cellular O_2_ requirements in the Sherpa (Murray et al., 2018), as supported by enhanced mitochondrial coupling efficiency (Horscroft et al., 2017). These metabolic adaptations were associated with the putatively advantageous allele of *PPARA* (Horscroft et al., 2017), previously reported as an adaptive target in Tibetans (Simonson et al., 2010). In stark contrast to Sherpas, lowlanders demonstrated depletion of skeletal muscle PCr and ATP alongside a sharp rise in oxidative stress markers with ascent (Horscroft et al., 2017). While there was evidence of a shift away from FAO through suppression of a PPARα target (carnitine palmitoyl transferase 1 B) and enhanced oxidative coupling efficiency, lowlanders also displayed evidence of incomplete FAO through an increase in long-chain acylcarnitines: total carnitine ratio (Horscroft et al., 2017), potentially resulting in production of harmful lipid intermediates (Koves et al., 2008).

Insight into metabolic acclimatization to high altitude has also been gleaned from metabolomics analysis of placenta collected at 3100 m following C-section or vaginal delivery from women of sea-level ancestry living at high altitude (Tissot van Patot et al., 2010). In contrast to sea level, labor at altitude generated greater ATP, ATP/ADP ratios and higher PCr in the absence of large changes in glucose, lactate, or free amino acids. This shift in metabolites occurred alongside evidence of decreased oxidative stress, lower lipid peroxidation and increased antioxidant capacity. Together, these changes implied that metabolic adaptation had occurred in response to maternal hypoxia at high altitude during pregnancy. This led to a blunted response to the hypoxia-induced metabolic stress of labor, with less reliance upon anaerobic glycolysis or protein catabolism to maintain energetic homeostasis (Tissot van Patot et al., 2010).

Metabolic signals have also been identified at the systemic level in response to high-altitude exposure. In the largest of these studies to date, metabolomic and lipidomic analyses were conducted on plasma obtained from 198 subjects at baseline and across four locations on the ascent to Everest Base Camp (5,300 m). Metabolites undergoing progressive changes with ascent were identified. Increasing lactate and decreasing glucose pointed towards increased reliance upon anaerobic glycolysis. This shift occurred alongside evidence of fat store mobilization, with decreasing triglycerides associated with *de novo* lipogenesis and increasing levels of free fatty acids such as palmitic, linoleic, and oleic acids (O’Brien et al., 2019).

Increased reliance upon glycolysis with high-altitude acclimatization is supported by other metabolomics studies, which demonstrate raised skeletal muscle glycolytic intermediates in lowlanders (Horscroft et al., 2017), raised circulating lactate (Tissot van Patot et al., 2009) and induction of erythrocyte glycolysis (Liu et al., 2016; Sun et al., 2016). Erythrocyte glycolytic pathways are tightly linked to O_2_ delivery, with raised sphingosine 1-phosphate (S1P) and phosphorylation of AMP-activated protein kinase both contributing towards hypoxia-induced 2,3-bisphosphoglycerate (2,3-BPG) (Liu et al., 2016; Sun et al., 2016), a negative allosteric regulator of Hb-O_2_ binding affinity, thus facilitating O_2_ release (Chanutin and Curnish, 1967). The induction of erythrocyte 2,3-BPG was shown to persist throughout prolonged (16 day) stay at altitude (D’Alessandro et al., 2016; Liu et al., 2016) and one week after descent (D’Alessandro et al., 2016).

Metabolomics has also been applied in the context of high-altitude pathology, as discussed in Section 5, with the aim to identify circulating biomarkers. In comparison to control subjects, those suffering from HAPE (10 per group) demonstrated distinct metabolic profiles, including increases in a number of amino acids (such as valine, lysine, and isoleucine), decreased glucose, and low density lipoproteins (Luo et al., 2012). Next research steps should aim to determine what physiology and biomarkers are common and perform research to understand the underlying mechanism.

While relatively few high-altitude studies have employed metabolomics techniques, current evidence suggests it is an effective approach for identifying metabolic signals of adaptation and acclimatization to high altitude. It provides the most insight when combined with functional measures to elucidate mechanisms associated with these signals, as demonstrated by Horscroft et al. (2017), Sun et al. (2016), and Liu et al. (2016).

### 6.3 Multi -Omics in Other Extreme Environments

While high altitude is the main environment in which humans encounter hypoxia, multi -omic tools can and have been applied to other extreme environments where humans may face the physiological challenge of low O_2_. One such instance is in diving populations, as exemplified by the Bajau people of the Philippines who are known for their underwater breath-holding abilities. The Bajau people spend an average of 60% of their work day underwater, and undergo hypoxic states during breath holds (Schagatay et al., 2011; Ilardo et al., 2018). Ilardo et al. (2018) identified genetic variants under positive selection within the Bajau that corresponded with larger spleen size, potentially conferring a physiological advantage during dives by providing a larger reservoir of red blood cells.

Further multi -omic work is being conducted in the final human frontier, where humans may encounter hypobaric and potentially hypoxic conditions during space travel. Currently, on-board the International Space Station (ISS), great care is taken to maintain a steady partial pressure of O_2_ near to that at the Earth’s sea-level, but explorers conducting extravehicular activity may be exposed to hypobaric hypoxic conditions (Norcross et al., 2015). Despite the normal O_2_ levels on-board the ISS, an integrated -omics study of an astronaut during a one-year mission on-board the ISS revealed changes in the expression pattern of genes that have been implicated in hypoxia in rodent models (Garrett-Bakelman et al., 2019). Furthermore, the ambient carbon dioxide (CO_2_) concentration in space is in excess of normal atmospheric conditions (0.7 versus 0.03%) (Matty, 2010; Cronyn et al., 2012). Studies are being conducted to understand the health impact of elevated CO_2_ on astronauts and the role of genetics in individual variability in response to elevated CO_2_ in space flight (Laurie et al., 2017). While the space travel environment may differ from high altitude, hypoxia and hypercapnia remain key concerns in maintaining astronaut health, and multi-omics analyses may provide insight into how to counteract environmental stressors (Schmidt and Goodwin, 2013; Beheshti et al., 2018).

## 7 Conclusion

Humans and other animals have experienced hypoxia across various time scales. This synthesis of existing knowledge of hypoxia responses across time domains integrates information from comparative animal and human studies and explores disease consequences for modern humans. Findings and perspectives across each of these domains contribute to unique and promising future directions for evolutionary and clinical hypoxia research. As tools and techniques become more sophisticated, ongoing and future studies in genomics, epigenomics, other -omics, and environmental/clinical phenotypes measured across species and across the lifespan must be integrated to fully understand how the challenge of hypoxia impacts various physiological systems.
